# Height, but not binding epitope, affects the potency of synthetic TCR agonists

**DOI:** 10.1016/j.bpj.2021.10.022

**Published:** 2021-11-06

**Authors:** Kiera B. Wilhelm, Shumpei Morita, Darren B. McAffee, Sungi Kim, Mark K. O’Dair, Jay T. Groves

(Biophysical Journal *120*, 3869–3880; September 21, 2021)

In the Supporting Material, Figure S3 did not display correctly, and Figure S6 was missing. The Supporting Material has been updated to correct those errors.

**Figure S3:**
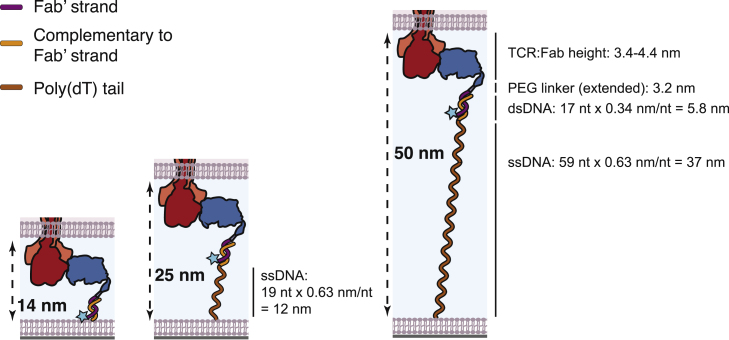
Approximation of intermembrane space allowed at binding events between Fab’-DNA and TCR, assuming oligonucleotides can fully stretch if needed. The intermembrane space for each thiol-DNA tether was estimated using the structure of H57 Fab bound to TCR (PDB: 1NFD), the estimated length of the PEG linker, the height of a double stranded DNA base pair, and the height of a single stranded DNA nucleotide. (original)

**Figure S6:**
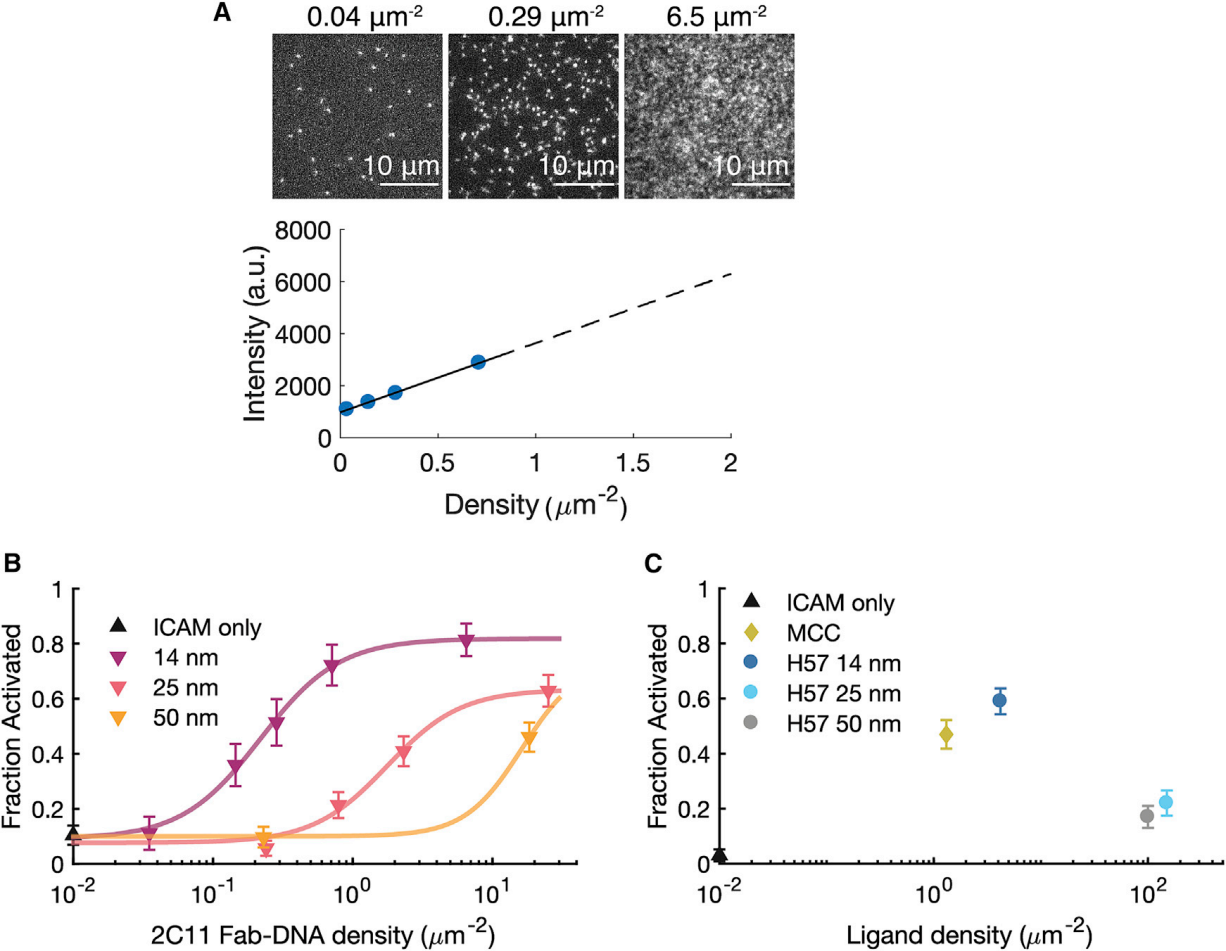
T cell activation as a function of ligand density for assayed ligands. (A) The density of ligand on the bilayer is determined by TIRF intensity. The total intensity and particle density are measured at densities for which particles are countable. The density-intensity calibration curve is then extrapolated to determine the density of ligand on high-density bilayers. (B) NFAT titration curves for 2C11 Fab’-DNA with varied tether lengths also show decreased ligand potency with increased tether length. The inflection points for all three constructs roughly match those for the corresponding H57 constructs. (C) Even at very high (∼100 μm^-2^) density, H57 Fab’-DNA constructs that allow up to 25 nm and 50 nm intermembrane space do not fully activate T cells compared to the 14 nm Fab’-DNA and pMHC controls, though they do activate significantly above the ICAM only negative control. Of note, cells in the experiment shown had a low maximal fraction of cells that activated in response to short ligands (∼0.6 compared to ∼0.8), which may relate to why the fraction activated, especially from the medium tether ligand, is low compared to the data set shown in panel (B) and Fig. 4D. n > 50 cells for all conditions in (B) and (C). (original)

